# The C‐terminal head domain of *Burkholderia pseudomallei* BpaC has a striking hydrophilic core with an extensive solvent network

**DOI:** 10.1111/mmi.14953

**Published:** 2022-07-01

**Authors:** Andreas R. Kiessling, Sarah A. Harris, Kathleen M. Weimer, Geoffrey Wells, Adrian Goldman

**Affiliations:** ^1^ Astbury Centre for Structural Molecular Biology School of Biomedical Sciences, University of Leeds Leeds UK; ^2^ Astbury Centre for Structural Molecular Biology, School of Physics and Astronomy University of Leeds Leeds UK; ^3^ IGBMC, School of Life and Health Sciences University of Strasbourg Strasbourg France; ^4^ UCL School of Pharmacy University College London London UK; ^5^ MIBS, Faculty of Biological and Environmental Sciences University of Helsinki Helsinki Finland

**Keywords:** bacterial adhesin, bacterial outer membrane proteins, *Burkholderia pseudomallei*, melioidosis, protein conformation, Type V secretion systems, β‐sheet

## Abstract

Gram‐negative pathogens like *Burkholderia pseudomallei* use trimeric autotransporter adhesins such as BpaC as key molecules in their pathogenicity. Our 1.4 Å crystal structure of the membrane‐proximal part of the BpaC head domain shows that the domain is exclusively made of left‐handed parallel β‐roll repeats. This, the largest such structure solved, has two unique features. First, the core, rather than being composed of the canonical hydrophobic Ile and Val, is made up primarily of the hydrophilic Thr and Asn, with two different solvent channels. Second, comparing BpaC to all other left‐handed parallel β‐roll structures showed that the position of the head domain in the protein correlates with the number and type of charged residues. In BpaC, only negatively charged residues face the solvent—in stark contrast to the primarily positive surface charge of the left‐handed parallel β‐roll “type” protein, YadA. We propose extending the definitions of these head domains to include the BpaC‐like head domain as a separate subtype, based on its unusual sequence, position, and charge. We speculate that the function of left‐handed parallel β‐roll structures may differ depending on their position in the structure.

## Graphical abstract

The structure of the highly negatively charged C‐terminal head domain of BpaC reveals a novel subtype of left‐handed parallel β roll. This expands the previously described YadA‐like head domains, which are primarily N‐terminal and positively charged. We identified an intricate buried solvent network within a left‐handed parallel β roll due to the hydrophilic nature of its core residues, expanding the structural universe of trimeric autotransporter adhesins. We speculate that the unusual charge is functionally important.

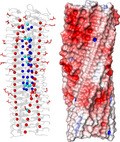

## 1 INTRODUCTION

*Burkholderia pseudomallei*, an aerobic Gram‐negative soil‐dwelling bacterial pathogen endemic in areas of Southeast Asia and Northern Australia, causes a wide variety of acute and latent diseases in humans. Acute infections (melioidosis causing a septic shock) can have mortality rates as high as 50%, and the bacterium is resistant to many front‐line antibiotics. The main route of infection is via aerosols after contact with an infected horse. Because of the low dose required for infection and the high propensity for aerosol formation, *B. pseudomallei* is considered a “class B” potential bioweapon (Wiersinga et al., 2006).

*B. pseudomallei* is an intracellular pathogen and escapes phagocytic digestion using a Type III secretion system (Gong et al., 2011). Once free, it spreads via intercellular fusion, thereby evading immune recognition (Burtnick et al., 2011). Type V secretion systems, including Type Vc trimeric autotransporter adhesins (TAAs) have been shown to be important virulence factors associated with adhesion and/or immune evasion. These include BoaA/B (Balder et al., 2010) and BpaA/C/D/E (Lazar Adler et al., 2015). *B. pseudomallei* can adapt to different ecological and host niches due to changes in gene and protein expression that alter factors like membrane composition, essential metabolism, and virulence (Duangurai et al., 2018); this may be important in understanding the different lengths and domain organizations of TAAs in *B. pseudomallei*. This also implies a complex interplay of different adhesins targeting a set of cells with high specificity: the adhesins expressed depend on signals from the environment, whether intracellular or extracellular.

The TAA BpaC was first extensively described in 2013 (Campos et al., 2013); of the nine predicted *B. pseudomallei* TAAs, BpaC is the only one associated with all of the three features involved in pathogenicity: macrophage survival, virulence, and serum survival (Lazar Adler et al., 2015). Studies have demonstrated BpaC adhesion to respiratory epithelial cell lines, establishing the importance of BpaC in the initial attachment and tropism of the pathogen (Lafontaine et al., 2014). BpaC thus represents a valid target for investigation as a potential drug target. The potential mechanisms of BpaC can be inferred from the roles reported for other TAAs: adherence, invasion, serum resistance, and biofilm formation (for a review, see Kiessling et al. [2020]). In general, TAAs bind many different partners such as parts of the extracellular matrix (Vaca et al., 2020), complement system down‐regulators like C4b‐binding protein (Hovingh et al., 2016), and specific receptors like the human carcinoembryonic antigen‐related cell adhesion molecule 1, which binds UspA1 (Conners et al., 2008).

**FIGURE 1:**
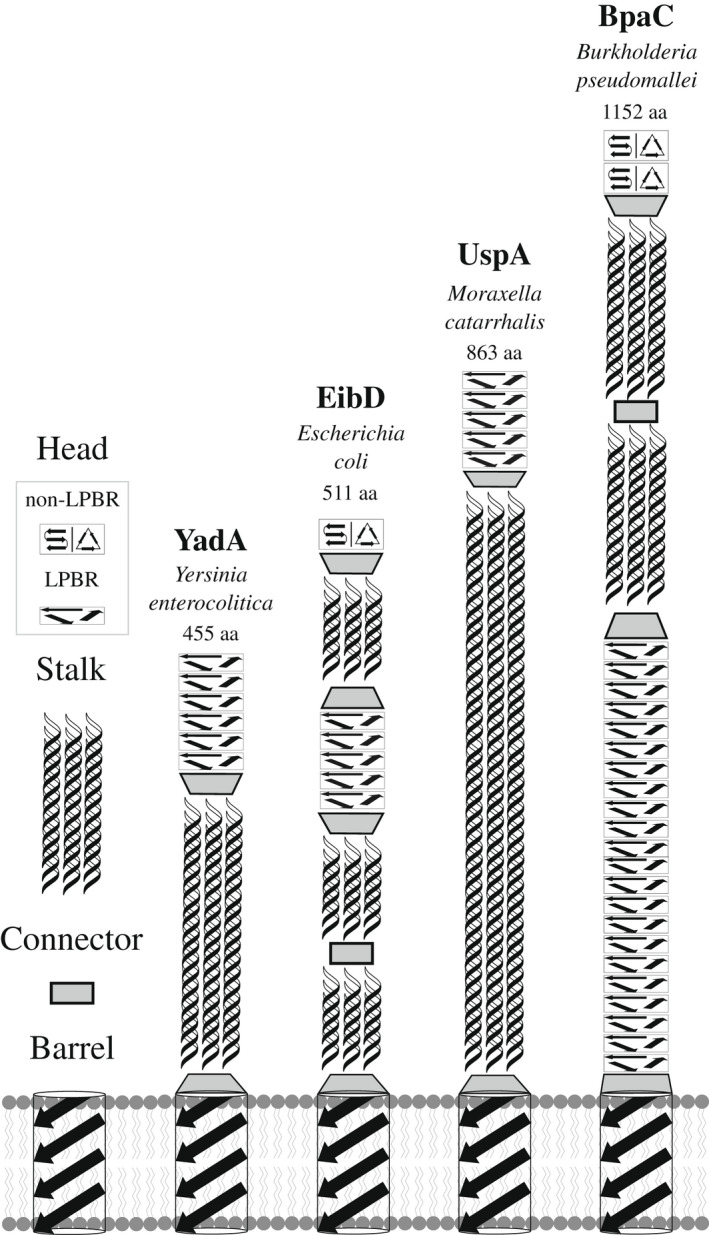
Schematic diagram of solvent‐accessible passenger domains of four different TAAs. Left: Structural modules in TAAs consist of β‐sheet rich head domains, coiled‐coil stalk domains, connector neck motifs, and the membrane‐anchoring β‐barrel domain. Head domain folds can be identified as either left‐handed parallel β‐rolls (LPBR) or non‐LPBR; with the latter predicted as either β‐meander or β‐prism motif (indicated by a split in the illustration). The different locations of the head domains are shown for the passenger domains of *Yersinia enterocolitica* YadA, *Escherichia coli* EibD, *Moraxella catarrhalis* UspA1, and *B. pseudomallei* BpaC. Connector domains show transition of coiled‐coil stalk domains to head domains or from the β‐barrel to the passenger domain. The length of each TAA is shown (aa).

The modularity of TAA domains in the solvent‐accessible region of the protein (the passenger domain), and the structural constraints imposed by their trimeric nature, enable the combination and diversification of protein function using a limited number of protein scaffolds of low sequence identity (Figure 1) with a general head‐stalk‐membrane β‐barrel architecture (Kiessling et al., 2020). Two examples of structurally‐diverse head domains are the left‐handed parallel β‐roll (LPBR) as in YadA (PDB: 1P9H; Nummelin et al., 2004) and the head domain of BadA (PDB: 3D9X; Szczesny et al., 2008) with two β‐prism motifs. Such well‐defined structural motifs and unique TAA sequence‐to‐structure connections have been used to predict at least parts of the structure of a TAA passenger domain through an expanding domain dictionary (a collection of sequence‐to‐structure relations in TAAs) and the bioinformatic program daTAA (Bassler et al., 2015; Hartmann et al., 2012; Szczesny & Lupas, 2008). For instance, the approximately 14‐long YadA head repeat has a canonical sequence motif of GxNSVAIGAxSxAx (Nummelin et al., 2004).

We solved the structure of an unusual region of BpaC to test homology model predictions of the C‐terminal region of the BpaC passenger domain. We show that it is an unusual LPBR with a hydrophilic “hydrophobic core,” analogous to the “N@*d*” motif identified by Hartmann et al. in TAA stalk regions (Hartmann et al., 2009). Intriguingly, and like “N@*d*” in EibD (Hartmann et al., 2009; Leo et al., 2011), this occurs close to the membrane anchor. In addition, the charge distribution of the LPBR is very different than YadA: it is highly negatively charged. We have identified other, similar headgroups in other members of *Burkholderia* using this new structural classification and speculate that change in charge may explain how BpaC is involved in the infection process of *B. pseudomallei*.

## 2 RESULTS

### 2.1 The structure of the C‐terminal head domain of BpaC

In our attempts to obtain a structure of BpaC, we noticed that the C‐terminal region was more stable and soluble than regions that included parts of either the N‐terminal head domain or the stalk domain. C‐terminal constructs stayed trimeric in SDS‐PAGE even after heating to 95 °C for 10 min in loading buffer (Figure S1) and could be concentrated to >130 mg ml^−1^. Having settled on BpaC^741–1054^ as the region to express, we used the engineered trimeric GCN4 leucine zipper (Hernandez Alvarez et al., 2008) to preserve the DAVNxxQL neck motif at the C‐terminal end by replacing the coiled‐coil that would be in the membrane β‐barrel in the *wt* structure. We solved the structure by molecular replacement using the C‐terminal head domain of BoaA (PDB: 3S6L; Edwards et al., 2011) as a model. There was a single molecule in the asymmetric unit, and the TAA trimer formed around the crystallographic threefold axis. The final R‐factors were 18.39%/21.71% (R_work_/R_free_) at a resolution of 1.4 Å and a total of 14 alternate conformations were built. The quality of the electron density is consistent with this resolution (Table 1) (Figure S2). The final model contains all of the residues in BpaC from position 14 in layer 22 to the end of layer 42 (residues 741–1021), followed by a long neck from E1022 to Q1054, the GCN4 anchor, with 282 water molecules, of which 42 are ordered in specific channels or the trimer core cavity.

**TABLE 1:** Diffraction data and refinement statistics for BpaC^741–1054^

Parameter	Value
PDB accession code	7O23
Data collection	
Space group	R32
Unit cell	
a, b, c (Å)	57.39, 57.39, 516.53
Resolution (Å)	44.79–1.40 (1.45–1.40)^a^
Reflections (observed/unique)	523,981/65682
R_merge_ (%)	5.43 (40.06)
Mean(I/σ[I])	7.13 (1.43)
Completeness (%)	99.53 (99.28)
CC_1/2_	99.8 (68.9)
Multiplicity	2.0 (1.9)
Wilson B value (Å^2^)	12.06
Refinement	
R_work_/R_free_ (%)	18.39/21.71
Protein/solvent/ligand atoms	2095/280/13
Average B (Å^2^)	
Protein	22.42
Solvent	29.11
Ligand	34.61
R.m.s.d., bonds (Å)	0.006
R.m.s.d., angles (°)	0.82
Ramachandran plot (%)	
Most favoured regions	98.47
Additionally allowed regions	1.53
Rotamer outliers (%)	0.93
Clashscore	4.21
Molprobity score	1.20

^a^ Values in parenthesis represent the highest‐resolution bin.

### 2.2 BpaC C‐terminal LPBR has been formed by expansion of a three‐layer motif

**FIGURE 2:**
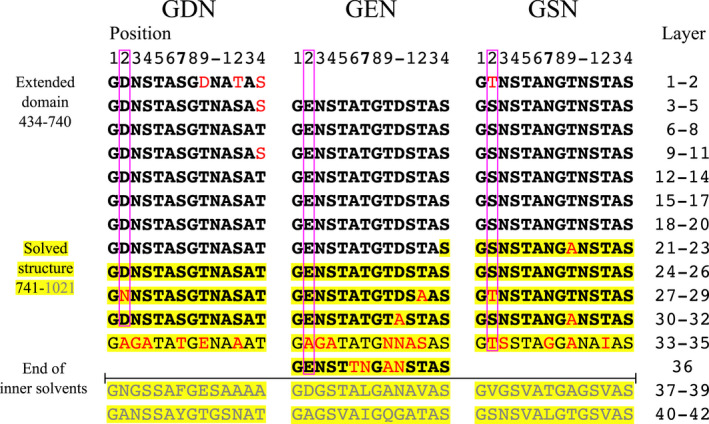
Alignment of the BpaC^434–1021^ region. 14‐residue long LPBR layer repeats are aligned to emphasize similarities between GDN/GEN/GSN families. Bold: identical copies of the consensus sequence in each family. Residues included in the solved structure have a yellow background and D/E/S@2 highlighted by magenta boxes. Residues that do not match the consensus sequence are colored in red. Layers 37–42 deviate too much from the GDN/GEN/GSN family assignment and are excluded (grey). The end of the inner solvent channel is indicated, as this correlates with the end of the GDN/GEN/GSN family assignments.

Sequence alignments show that BpaC^434–1021^ contains 42 14‐residue LPBR repeats, and intriguingly, it contains clear evidence (Figure 2) of a triplet‐LPBR expansion. In this region, layers 3–26 and 30–32 have almost identical repeats of a “GDN”‐“GEN”‐“GSN” three‐layer motif, while layers 1 and 2 are “GDN”‐“GTN”, and layer 36 is a GEN‐type. The structural consequences of this are discussed in what follows.

### 2.3 BpaC is an unusual left‐handed β‐roll with a primarily *hydrophilic* core

All three chains of the C‐terminal head domain of BpaC thread around a central hydrophilic core in a helical fashion with an average turn of 5.7° ± 0.6° for layers 23–41 and a rise of 4.8 Å ± 0.1 Å (the last one, from 1008–1021, has a turn of just 2.5°), calculated per turn, typical of other LPBRs. From layer 23 to 42, the total twist is 103.8°, and the total rise is 81.5 Å (Table S1). In our numbering below, we refer to layer numbers from the full C‐terminal head structure: The first layer in the solved structure is number 23.

**FIGURE 3:**
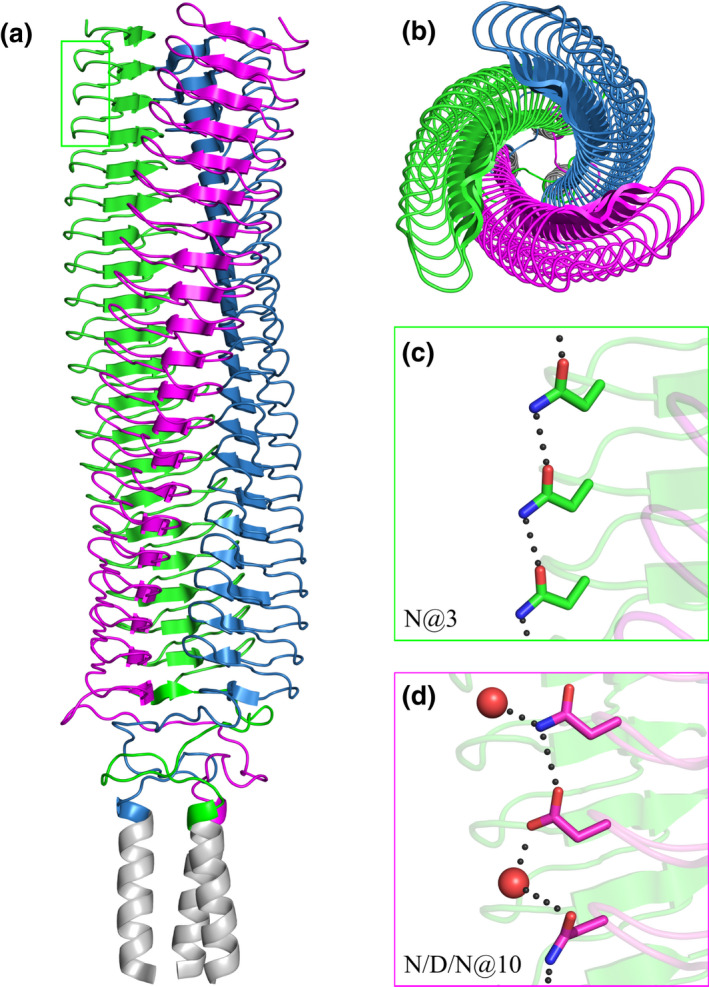
Overall view of the C‐terminal head domain of BpaC^741–1054^. The structure covers 20 of the predicted 42 LPBR head repeats, each layer consisting of 14 amino acids. The three monomers are colored magenta, blue, and green, apart from the added GCN4 leucine zipper (grey). (a) Side view. (b) Top view from the N‐terminus. (c) N@3 in the first 10 repeats form a continuous network of stabilizing hydrogen bonds (only three shown). (d) N/D/N@10 also forms a network of stabilizing hydrogen bonds with interspersed solvent molecules (red spheres).

Despite having a canonical LPBR structure (Figure 3a,b), there are a number of unique features. First, the presence of asparagine at position 3 within a 14‐residue repeat (N@3) leads to a long chain of hydrogen bonds, completely conserved to the N‐terminus of this domain, in the first 10 layers of our structure (Figure 3c). We use X@m to denote single residue code X at position m (m = 1–14) in the 14‐residue repeats. Next to this in the neighboring monomer is a three‐layer repeat motif of N/D/N@10 (Figure 3d) also forming stabilizing hydrogen bonds down the spine of the structure.

**FIGURE 4:**
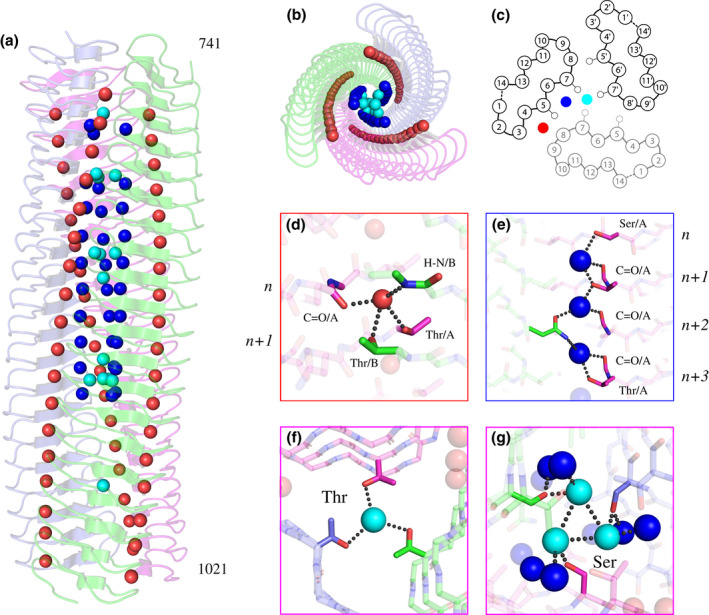
Solvent channels and interactions in BpaC^741–1021^. Water molecules are color‐coded: outer (red), inner (blue), and central (cyan). The three monomers are shown in magenta and blue, with hydrogen bonds in black dots. (a) Side view of the different solvent channels of BpaC^741–1021^ from N‐ to C‐terminus. (b) Top view of BpaC^741–1021^ showing the different solvent channels. (c) Schematic of a 14‐residue layer of BpaC^741–1021^ indicating the location of the various solvent channels alongside the Cβ atoms of residues 5 and 7 to help distinguish the different channels. (d) Outer solvent channel: stabilizing chiefly by interactions between backbone atoms in layer “*n*“, and the γOH of T@5 in layer “*n+1*” between two monomers. (e) Inner solvent channel: stabilized by interactions of hydrophilic sidechains S/T/N@7, and supported by backbone carbonyls. The γOH of S/T@7 connects to the inner solvent channel from monomer “A” (cyan spheres) while the interactions of N@7 contribute from the adjacent monomer “B” (green). (f) The central solvent molecule, with the only interaction from an adjacent T972@7 γOH. (g) The central solvent channel stabilized by a S846@7 γOH and further connections to the inner solvent channel.

Second, there are three identifiable solvent networks, which we have termed “outer,” “inner,” and “central” (Figure 4a–c). The location of these channels can be defined by their relative location within the LPBR repeats: The “outer” solvent molecules, present in *all* LPBRs (Figure S3), had not been identified by previous authors including ourselves. We describe them for the first time here: They form an ordered part of the structure between residues 3–4‐5 within the 14‐residue layers and are 4.8 ± 0.3 Å apart. The “inner” water molecules, situated between residues 5–6‐7, are 4.6 ± 0.2 Å apart and completely buried; the “central” solvent molecules are closer to the threefold axis, near the sidechain of residue 7/14, and some, but not all, are hydrogen‐bonded to the inner channel.

**FIGURE 5:**
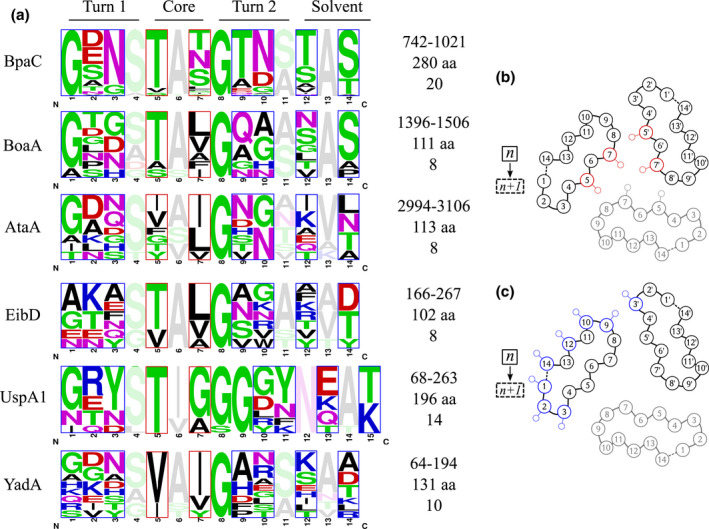
Residue frequencies for TAA left‐handed parallel β‐roll repeats, in which the height represents the frequency of residues in each layer. The G@8 is completely conserved. Each layer consists of 14 residues except for the mostly 15‐residue repeats in UspA1, and rare loop insertions or deletions that were excluded prior to logo creation (see Figure S4). These occur between Pos14/14 of layer “*n*” and Pos1/14 of layer “*n + 1*”. (a) Sequence logos of residue frequencies. Amino acids are colored by side‐chain properties. Residues that face into the chain interior are shown transparently for better visibility. (b) Cβ positions of amino acids contributing to core interactions are boxed in both frequency plot and sketch (red). (c) Solvent‐facing Cβ positions are highlighted in both plot and sketch (blue). There is one exception to the completely conserved G@8: UspA1 S105 (PDB: 3PR7; Agnew et al., 2011). We ascribe this to an error in the sequence or the structure of the protein, as the ϕ and φ angles are disallowed for serine and there is no sidechain density even at Cβ in the 2*F*_*o*_ *‐F*_*c*_ map (data not shown).

In BpaC, the outer solvent channel is typically formed by four H‐bonds/dipole interactions: two conserved interactions, one to the monomer “A” backbone carbonyls of position 3/14 of layer “*n*” and the other from the monomer “B” backbone amide of position 9/14 of layer “*n*,” and the last two found most often in BpaC, from the sidechain γOH of monomer “A” and “B” position 5/14 in layer “*n + 1*” (Figure 4d). Position 5 is almost always threonine in BpaC (Figure 5), providing extra stability to this interaction in comparison with YadA. The interaction clearly provides stability between monomers, and between layers in an individual monomer.

More surprising, however, are the “inner” and “central” chains of buried water molecules, which form because the BpaC core is uniquely hydrophilic in 15/20 of the LPBR layers, composed of repeating units of N/S/T@7 (Figure 2). H‐bonds from the γOH of S/T@7 to the inner water serve as a bridge between layers, which are supported by intramonomer H‐bonds from the carbonyl oxygen on (conserved) A@6 in layers “*n + 1*” to “*n + 3*” (Figure 4e). The asparagine layers in addition bridge between monomers, so the sidechain amide of N@7 in layer “*n + 2*” of monomer “B” hydrogen bonds to the inner water, which in turn hydrogen bonds to monomer “A” A@6 backbone carbonyl and in some cases also to monomer “A,” level “*n + 3*” of the γOH of T@7. The chain of hydrophilic interactions spans across layers and between monomers. With the exception of the special solvent network in layers 35 and 36 with G@7 and N@7, central solvent molecules are H‐bonded to the γOH of S/T@7, either sharing a central molecule or having one solvent molecule per residue (Figure 4f,g).

**FIGURE 6:**
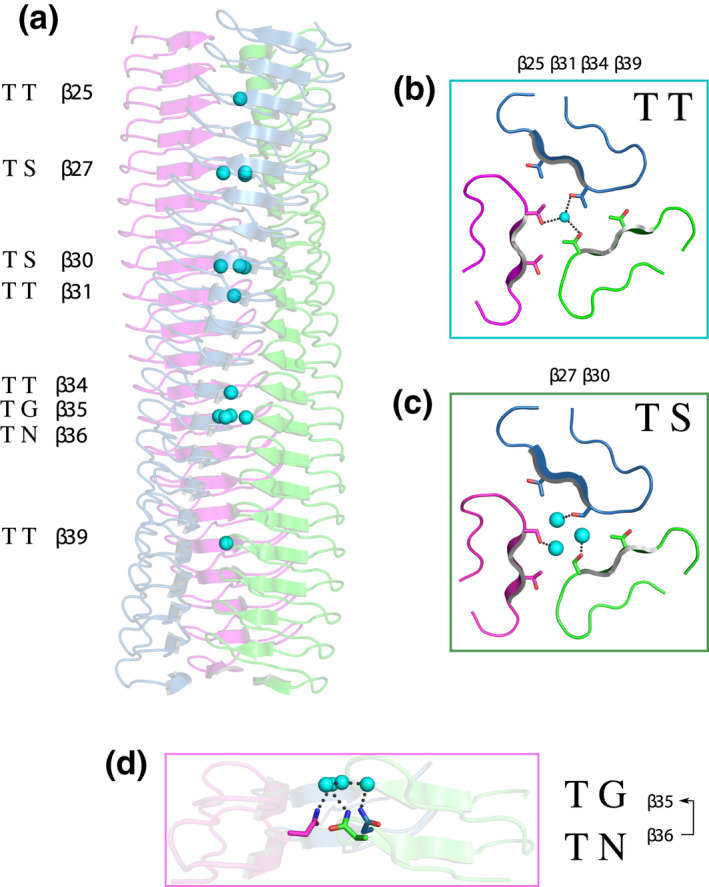
Varying arrangement of central solvent molecules in BpaC^741–1021^. (a) Side view of BpaC^741–1021^ showing LPBR layers containing solvent molecules (cyan spheres) within the trimer core. Layers with shown motifs are numbered; motifs without solvent molecules are not included in this Figure. (b) Top view of a T@7 layer. Hydrogen bonds (black) are indicated between three γOH sharing a single solvent molecule. (c) Top view of hydrogen bonds is shown between three S@7 γOH and three solvent molecules. (d) Side view of hydrogen bonds displayed between three N@7 δNH and four solvent molecules arranged in a tetragonal arrangement in the layer above. The missing sidechain of the G@7 creates a large cavity for these solvent molecules.

Finally, the core of the molecule around the threefold axis is completely hydrophilic in 15 of the 20 layers in the solved structure (23–36 and 39), as well as all of the layers 1–22 due to N/S/T@7, supported by hydrophilic residues at T/S@5 (Figure S5). Layers 23, 26, 29, and 32 (N@7) have, we believe, an unusual arrangement in which, stochastically, the δΝΗ of one of the three asparagines points into the center, enabling a hydrogen‐bond network around the three‐fold (Figure S5, T N). At layers 35–36 (G@7, N@7), a tetrahedral arrangement of water molecules forms, with the increased space at G916 allowing one water molecule on the three‐fold axis hydrogen‐bonded to a water molecule attached to each N930 in the layer below (Figure 6d). Only the very C‐terminal end of the LPBR (layers 37–38 and 40–42) have the hydrophobic residues usually found at positions 5 and 7 of an LPBR (Figure 5, YadA). The hydrophilic core includes 14 “central” buried water molecules distributed over 7 layers (Figure 6a): five on the threefold axis (Figure 6b,d) associated with layers 25 (T776), 31 (T860), 34 (T902), 36 (N930), and 39 (T972) and nine (one per monomer per layer) ≈ 2 Å from the threefold axis associated with layers 27 (S804), 30 (S846), and 35 (G916) (Figure 6c,d). This *hydrophilic* core coupled with very high stability (Figure S1) is one of the unique structural features of the BpaC head.

We performed molecular dynamics (MD) simulations to verify our observations of the described novel solvent patterns in BpaC—specifically the inner solvent channel and the central solvent molecules. Hydration densities approximately 7 times that of bulk water were found to correlate with most of the experimentally observed solvent molecules (Figure S6). Our hydration density maps confirm the presence of the inner solvent channel from layers 23–24 to 35–36, even filling in solvent molecules not visible in our X‐ray structure model between layers 23–24, 24–25, and 26–27 (Figure 4a for comparison). Additionally, the layers containing S@7 and T@7 that do not have central solvent molecules in the X‐ray structure model (Figure S5, layers 24, 28, and 33) have a visible density in the hydration density maps. On this basis, a reasonable assumption would be that the solvation trend for the central and inner solvent channel in the crystal structure can be extrapolated to the rest of the C‐terminal head domain (residues 434 to 740). The MD simulations also support our final refinement strategy of not building any possible ions for locations that would have had a viable amount of coordination sites for them. In essence, no significant replacement of solvent molecules by potassium ions occurred during the simulation.

### 2.4 The electrostatic charge on an LPBR domain reveals a new head domain subcategory

**FIGURE 7:**
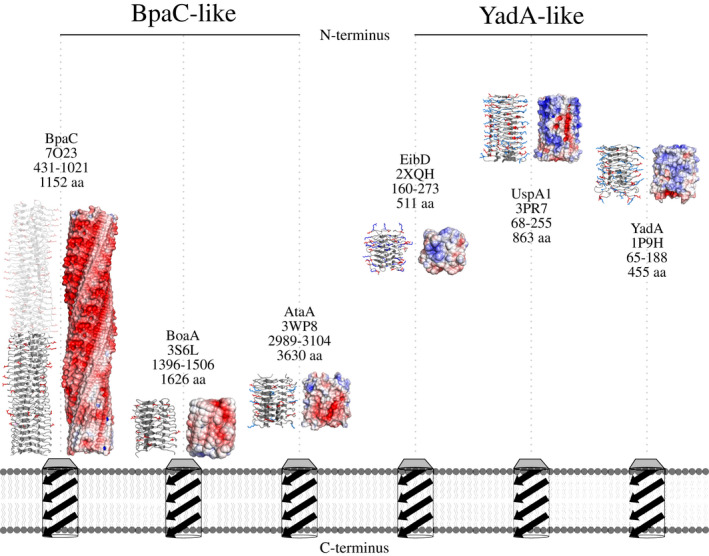
Surface charge distribution of selected LPBR head structures. The structures were trimmed to contain just the LPBR layers to show the distribution of solvent‐accessible side chain charges (APBS plugin, PyMOL). Structures are sorted by surface charge (negative to positive, red to blue). Structures are labeled with name, PDB ID, residues used for APBS map, and overall size of the whole TAA. The positions of the head domains relative to the membrane and TAA termini are indicated. Models not to scale. BpaC is divided into a known structure (741–1021, grey) and model inferred by the identity of repeats (431–740, transparent). LPBR structures are divided into BpaC‐like and YadA‐like depending on their relative position and their surface charge profile.

The other unique structural feature is the negative electrostatic charge of the BpaC head (Figure 7). This is due to the presence of D/E@2 on the outside of the trimer in the “GDN” and “GEN” layers, and the aspartate in N/D/N@10 (Figure 2). The D/E@2 within a single monomer are next to the chain of hydrogen bonds running down the spine of BpaC created by N@3 (Figure 3c) from layer 1–31 (see above), while the D@10 is relatively close (Figure 3d) in a neighboring monomer in the “GEN” layers (Figure 2) and held in position by N@10 in the “GDN” and “GSN” layers. Unsurprisingly, the full‐length BpaC head (Figure 7, transparent model), has rows of negatively‐charged residues forming a distinct helical pattern on the surface of BpaC, possibly of functional significance.

Unlike all other YadA‐like head domain structures (Figure 7), BpaC only has negatively charged residues on its surface. Furthermore, the electrostatic surface charge in YadA‐like head domains seems to correlate with the relative position of the head in the passenger domain (Figure 7): the closer the head domain is to the N‐terminus (i.e., membrane distal) the higher the ratio of positively‐to‐negatively charged residues became (Table 2). BpaC is thus the archetype for a new subcategory of negatively charged LPBR membrane‐proximal head domains. These differences can be seen clearly in the LBPR frequency plots (Figure 5). Focussing on the charged solvent‐accessible residues (Table 2) shows that BpaC and BoaA exclusively have negatively charged residues, while the others have a mix of positively and negatively charged residues, and this is especially so for LPBRs toward the more N‐terminal end of the protein (Figure 7). The pIs of the solved LPBR structures demonstrate this clearly: the pI of the LPBR is clearly correlated with its position within the passenger domain, with BpaC having a pI of about 2.3 and YadA of 9.2 (Table 2). EibD, located centrally, is the only one with an almost neutral pI.

**TABLE 2:** Number of charged residues in selected LPBRs

	BpaC	BoaA	AtaA	EibD	UspA1	YadA
Head position	742–1021	1396–1506	2994–3106	124–266	166–267	64–194
Length (aa)	1152	1626	3630	511	863	455
Positively charged	0	0	3	8	17	11
Negatively charged	34	3	6	7	14	10
Positive (%)	0	0	33	53	55	52
Negative (%)	100	100	67	47	45	48
Average pI	2.32	3.93	4.71	6.44	8.90	9.17

*Note*: Solvent‐accessible residues were counted and only the ones carrying a charge at pH 7 are listed. The overall size of the whole protein is given alongside the position of the head domain. The amount of positively and negatively charged residues per LPBR is given in % of total charged residues. pI value was estimated in IPC 2.0 (Kozlowski, 2021) using the residues of the head position as input. Reported is the average pI value for all results out of IPC 2.0.

### 2.5 Identification of a BpaC homolog from *Burkholderia oklahomensis* and the evolutionary relationship of TAA head domains

**FIGURE 8:**
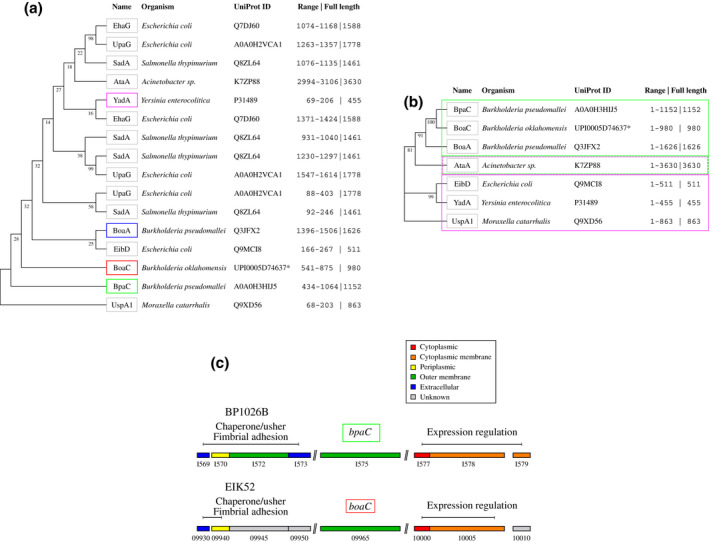
Evolutionary relationship of TAA LPBRs and genetic environment of *bpaC* and *boaC*. (a) Alignments of LPBRs with the BpaC (green) are produced based on the conserved G@8. A phylogenetic tree is shown with TAA names, organism name, UniProt ID, the range of residues included in the final alignment, and the total length of the TAA. “Adhesin YadA‐like” or BoaC (red) from *B. oklahomensis* is given with UniParc ID (*). The BoaA head domain which was used for molecular replacement for the structure in this publication is highlighted (blue). UspA1 is a separate branch, probably due to the unusual 15‐residue repeats. YadA as the canonical LPBR is highlighted to show the distance to BpaC (magenta). (b) Alignment of full‐length sequences focussing on TAAs that include the LPBR domains analyzed in the previous structural comparison. Clade assignment was split into BpaC‐like (green box with solid line) and YadA‐like (magenta box with solid line) supporting our previous subclassification evolving out of the structural comparison of LPBRs. AtaA is assigned to BpaC‐like and YadA‐like (dashed lines), as the full sequence contains both an N‐terminal and a C‐terminal LPBR head domain. (c) Genome island surrounding *bpaC* (green) and *boaC* (blue). Locus tag of genes adjacent to *bpaC* of *B. pseudomallei* 1026b and *boaC* of *B. oklahomensis* LMG 23618 is displayed in shortened form (BP1026B_X and EIK52_X). Localization of gene product is shown.

Similar domains found in multiple TAAs (e.g. LPBRs) can display high structural conservation despite variations in sequence identity and length. This has an impact on the accuracy of phylogenetic analysis and prompted us to refine our analysis to only include LPBR head domains with the fully conserved G@8. Fifteen sequences from well‐characterized TAAs across nine bacterial species were identified as possessing domains with the fully conserved G@8 required for LPBR heads and selected for subsequent phylogenetic analysis. All sequences in the initial analysis were aligned by the G@8 and trimmed using neck motifs as a domain boundary. The alignment of head domains was then used to build an HMM profile and perform an HMM search to identify novel head domains by their conserved G@8 for further analysis (Figure 8a).

Analysis of the head domains revealed a clade linking the BpaC head with a sequence from *Burkholderia oklahomensis*, “Adhesin YadA‐like” (UniParc: UPI0005D74637)—which we have named BoaC (o for *oklahomensis*)—like the one most closely related to BpaC. The “Hep‐Hag Family Protein” BoaA from *B. pseudomallei*, used for molecular replacement (PDB: 3S6L), is a more distant relative of BpaC. Full‐length BpaC and BoaC possess 78% identity, with 86% identity over the C‐terminal head domain. BoaC has a much shorter C‐terminal head domain with two separate deleted segments (BpaC equivalent of ΔA761‐N874 and ΔA920‐A949) that together correspond to 10 × 14‐residue LPBR layers (Figure S7). A further deleted region, likely a coiled‐coil segment, can be found at the N‐terminal end of the protein (BpaC equivalent of ΔS266‐A299). Using this information we extended the analysis to full TAA sequences but only included those which belong to the six LPBR heads included in the previous structural comparison (Figure 7). The resulting phylogenetic tree (Figure 8b) revealed clades that confirm our proposed differentiation into BpaC‐like head domains (BpaC, BoaC, BoaA) and YadA‐like head domains (EibD, YadA, and UspA1). AtaA is unusual because the full‐length sequence includes both an N‐terminal LPBR head domain (residues 110–265) and a C‐terminal head domain (residues 2989–3104), which is the likely reason for the branch position in between both categories. UspA1 has a separate clade in both trees as it contains an irregular 15‐residue repeat with an additional G@9 that likely impacted the alignment generation.

Further proof that *bpaC* and *boaC* are closely related is that they are both part of very similar pathogenic islands, unlike other TAAs included in this study: downstream of a chaperone‐usher pili assembly gene cluster, and upstream of a two‐component response regulator gene cluster, suggesting that both the pili and BpaC are involved in initial adhesion steps of the infection process in both organisms (Figure 8c).

## 3 DISCUSSION

### 3.1 BpaC ^741–1054^, the largest LPBR head structure so far, has an atypical surface charge and core

The structure reported here of BpaC^741–1054^ contains the highest number of LPBR repeats in an experimental structure and is the longest, at 89.5 Å. Its behavior, in crystallizing with the trimer arranged around a crystallographic 3‐fold axis, indicates that LPBRs are essentially rigid over at least 10 nm length. We estimate the size of the full‐length C‐terminal head domain to be about 20 nm, with a twist of about 260°. Using the domain annotations and our predicted homology models, the overall length of BpaC is about 55 nm including the anchor domain. BpaC (1152 residues) is thus at the lower medium‐sized end of the TAA spectrum compared to YadA (422 residues, ~20 nm; Hoiczyk et al., 2000) and BadA (3973 residues; Thibau et al., 2022); ~240 nm; Müller et al., 2011).

**FIGURE 9:**
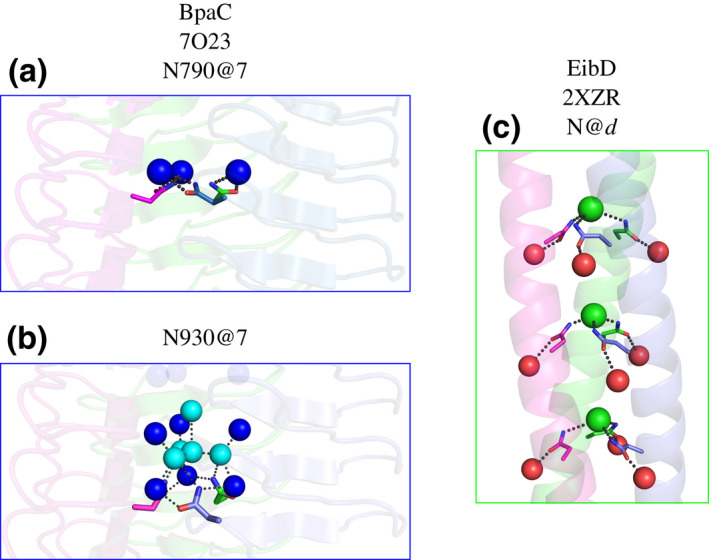
Comparison of N@*d* motif in EibD with BpaC N790 and N930 interactions—(a) Solvent interactions of the inner channel (blue spheres) are displayed with N790@7 as the example for other layers like this in BpaC. (b) A complex solvent network involving central solvent molecules (cyan spheres) and the inner channel (blue spheres) are being supported by N930@7 pointing toward the layer above which has G@7 that creates a cavity for these solvent molecules to fill in. (c) The N@*d* motif in the coiled‐coil of EibD (2XZR; Leo et al., 2011) sequesters chloride ions (green spheres) and interacts with adjacent solvent molecules (red spheres).

The core of BpaC is very different from YadA (Figure 5). Instead of predominantly β‐branched *hydrophobic* residues (Nummelin et al., 2004) at positions 5 and 7, BpaC has T@5 almost exclusively as well as a number of hydrophilic residues with T/N/S/G@7. T@5 is common in other LPBRs, particularly BoaA, EibD, and UspA, but BpaC is the only example of an LPBR with hydrophilic residues at position 7. This leads to the hydrophilic core, complete with “inner” and “central” water molecules, and may explain why the 42‐residue BpaC repeat is maintained throughout the domain: all three layers are needed to stabilize the hydrophilic core. The arrangement is analogous to, but much longer than, the hydrophilic “hydrophobic core” found in the coiled‐coil region of EibD (Leo et al., 2011) and described as “N@*d*” by Hartmann et al. (Figure 9) (Hartmann et al., 2009). We speculate that these structural repeats can be used to adapt to environmental changes by addition or subtraction of individual 14‐residue long layers within the head domain. This allows modulation of the overall length of the TAA and changes in the binding surface of the head domain. Because of the strong protein sequence similarity, the DNA tandem repeats are very similar, possibly providing a mechanism for rapid headgroup expansion and bacterial adaptation (Zhou et al., 2014).

In addition, there is a distinct trend in the charge on LPBRs: those that are N‐terminal, distal from the membrane, like YadA, are more positively charged, and those that are C‐terminal, close to the membrane, are negatively charged (Figure 7). Indeed, the pI of the BpaC C‐terminal head, as estimated by IPC 2.0 (Kozlowski, 2021), is around 2.3, whereas the pI of the N‐terminal head domain of YadA is around 9.2. We, therefore, propose that YadA‐like head domains can be subdivided into two categories: YadA‐like head domains (more N‐terminal, positively charged, hydrophobic core) and BpaC‐like head domains (more C‐terminal, negatively charged) as shown in Figure 7.

### 3.2 Potential functional differences between LPBRs

What is the biological significance of this large difference in charge? We suggest that it enables LPBR head domains to bind different cellular and extracellular matrix components. The solvent‐facing side chains are modulated to accommodate changing environmental conditions with a highly‐conserved fold. YadA, for instance, binds collagen (El Tahir & Skurnik, 2001), and positively‐charged residues have been shown to be important in this interaction (Nummelin et al., 2004), while UspA1/2 binds laminin (Tan et al., 2006). We speculate that BpaC C‐terminal LPBR, close to the membrane surface, may interact with the positive ions trapped in the lipopolysaccharide layer. Conversely, the BpaC N‐terminal β‐prism head due to its hypothesized similarity to the BadA head, which binds to fibronectin (Kaiser et al., 2008), would interact with similar intracellular ligands as *B. pseudomallei* is an intracellular pathogen. There are other examples of LPBRs with different charge properties: the predicted model of the N‐terminal LPBR of AtaA (Ishikawa et al., 2012) (residues 110 to 265, UniProt K7ZP88) has a positive electrostatic surface and mostly positively charged surface‐facing residues (Figure S8) as opposed to the structure of the C‐terminal LPBR, which is clearly negative (Figure 7). The N‐terminal part of AtaA plays a major role in the adhesion properties of this protein while the C‐terminal part contributes to the flexibility and toughness of the overall structure (Koiwai et al., 2016). A more mixed charge profile can be seen for the predicted model of the N‐terminal LPBR of BoaA (residues 180 to 437, UniProt Q3JFX2, Figure S8) which still differs from the exclusively negative surface charge of the C‐terminal head domain (Figure 7). The diversity of LPBRs is much greater than expected, and the presence of a hydrophilic core that is greater than 20 nm in length may have potential synthetic biology applications.

### 3.3 The homolog of BpaC in *Burkholderia oklahomensis* enables microbiological studies in a BSL‐2 environment

Elucidating the function of *B. pseudomallei* proteins is challenging, as it is a biosafety level 3 (BSL‐3) pathogen, which—appropriately—limits research access and the ability to make mutations to study function (Cheng, 2010). Using our G@8 alignment method for classifying LPBR repeats into N‐terminal YadA‐like head domains and C‐terminal BpaC‐like head domains, we identified a homolog, BoaC in *B. oklahomensis*, a BSL‐2 pathogen with 86% identity in the head sequence and 78% identity over the full‐length protein. As TAAs often have low sequence similarity despite high structural conservation, these proteins are likely true functional homologs despite the presence of some insertions and deletions. *B. oklahomensis* should thus provide an effective way to study the function of BpaC in a near‐native host environment, especially as both the upstream and downstream gene environments for *bpaC* and *boaC* are very similar (Table 3, Figure 8b). This strongly supports the functional connection and transferability of scientific findings between these two proteins.

**TABLE 3:** Overview of genes adjacent to *bpaC*

Locus tag	Refseq ID	Gene product	Function/GO term
BP1026B_I1569	WP_004521426	Fimbrial subunit	Fimbrial‐type adhesion domain
BP1026B_I1570	WP_004527078	Fimbrial chaperone protein	PapD‐like
BP1026B_I1572	WP_004527078	Usher protein	PapC‐like
BP1026B_I1573	WP_004554117	Type‐1 fimbrial protein	Pilin (type 1 fimbria component)
**BP1026B_I1575**	**WP_014696818**	**BpaC**	**Pathogenesis**
BP1026B_I1577	WP_004193126	DNA‐binding response regulator	DNA‐binding response regulator
BP1026B_I1578	WP_004531338	Two‐component regulatory system, sensor kinase protein	Phosphorylation, signal transduction
BP1026B_I1579	WP_004550403	EAL domain‐containing protein	EAL domain, signalling protein

*Note*: Gene annotation of adjacent functional clusters of *bpaC* that are either selected as part of the predicted pathogenicity island (I1569‐I1575) or the possible association is inferred by literature reference (I1577‐1579).

BpaC in bold as the gene around which we searched.

In conclusion, we have identified the first hydrophilic LPBR core: N/S/T@7 in the C‐terminal LPBR head domain of BpaC. Its primarily negative surface charge (D/E@2 and D@10) distinguishes it from the mainly positive charged N‐terminal head domain of YadA and so these represent two different classes of LPBR heads with different functional roles. We have also identified a close homolog of BpaC in *B. oklahomensis*, a BSL‐2 strain, which we propose to be named BoaC to reflect the close relationship between these two proteins. This homolog may help future investigations of the function of BpaC, identification of its binding partners, and the studies of the role of different charges on head domain function.

Finally, we note that, at the time of writing, the UniProt entry for BpaC (A0A0H3HIJ5) has an AlphaFold model (AF‐A0A0H3HIJ5‐F1; Jumper et al., 2021) that describes the full‐length trimeric BpaC as a monomer that folds back into the membrane anchor. TAAs are obligate trimers and essentially linear over all distance scales examined, so this monomeric model is misleading and cannot be used to inform structural or functional studies.

## 4 EXPERIMENTAL PROCEDURES

### 4.1 Expression and Purification of the BpaC C‐terminal head domain

We used a “*divide‐and‐conquer”* approach to split BpaC up. Because TAAs are repetitive and modular, they can be split into smaller segments, even in the middle of domains: their modular nature predefines logical construct start and endpoints. By making a series of constructs, we identified that the part of the gene encoding a fraction of the C‐terminal head domain of BpaC ranging from S741 to Q1054 (BpaCCHead, UniProt: A0A0H3HIJ5) was expressed in high yield and soluble. We amplified this region from synthetic DNA along with a one‐sided GCN4 anchor fragment (denoted RearGCN4 in Hernandez Alvarez et al. [2008]) from the pIBA‐GCN4tri vector (gift from Dirk Linke, University of Oslo, Norway) and the backbone of the pET28‐a vector (Novagen) (Table S2) to create the BpaC^741–1054^ fragment. RearGCN4 extends the coiled‐coil segments and increases domain stability (Hernandez Alvarez et al., 2008) and, in our case, replaces the coiled‐coil inside the β‐barrel transporter domain.

Fragments were amplified using Q5® High‐Fidelity 2X Master Mix (New England Biolabs) with the annealing temperature calculated from OligoCalc (Kibbe, 2007). Analysis and purification of each PCR reaction product were performed using a 1% agarose gel stained with SYBR® Safe DNA gel stain (Invitrogen) followed by the Macherey‐Nagel™ NucleoSpin™ Gel and PCR Clean‐up Kit (Fisher Scientific) according to the manufacturer's protocols. Purified PCR products were then combined by a 3‐part Gibson assembly with NEBuilder® HiFi DNA Assembly. Heat‐shock transformation of a ligated vector into chemically competent One Shot® OmniMAX™ *Escherichia coli* cells (Invitrogen™, Thermo Fisher Scientific) was performed according to the manufacturer's protocol and plated onto LB agar plates with Kanamycin antibiotic selection. Amplification and purification of plasmid DNA using NucleoSpin Plasmid Mini Kit (Macherey‐Nagel) were done following the manufacturer's instructions. The target sequence was confirmed by sequencing (Mix2Seq kit, Eurofins Genomics). pET28a‐BpaC^741–1054^ was then transformed into *Escherichia coli* BL21(DE3) cells and plated onto an LB agar plate with Kanamycin for selection.

Expression of BpaC^741–1054^ in LB media was induced at an OD600 value of 0.6 at a final concentration of 750 μM Isopropyl‐β‐D‐thiogalactopyranoside for 4 h. Cells were harvested by centrifugation (4000 × g for 20 min at RT) and stored at −20 °C until further use. Frozen cells were thawed on ice in 20 ml buffer *A* consisting of 50 mM NaH_2_PO_4_ pH 8, 300 mM NaCl, and 10 mM Imidazole pH 8. Cells were lysed using sonication and a further 15 ml of buffer *A* was added before centrifugation (40,000 × g for 40 min at 7 °C). The supernatant was transferred to a 50 ml falcon tube containing Ni Sepharose© 6 Fast Flow resin (GE Healthcare Life Sciences) preincubated with buffer *A* and left on a tube roller for 15 min at RT. Resin and supernatant were then transferred to an Econo‐Pac® Chromatography column (Bio‐Rad). The flowthrough was collected and 20 column volume (CV) buffer *A* added to the column. Further wash steps consisted of 5 CV buffer *B* (buffer *A*, 30 mM Imidazole pH 8) and 10 CV buffer *C* (buffer *A*, 50 mM Imidazole pH 8). Elution of target protein was carried out using 8 CV buffer *D* (buffer A, 250 mM Imidazole pH 8). The purity of the sample was assessed by SDS‐PAGE. The protein was dialyzed into 2 L of 20 mM Tris–HCl pH 8, 150 mM NaCl using SnakeSkin™ Dialysis Tubing (10 K MWCO, Thermo Fisher Scientific) overnight at 7 °C. Protein was concentrated using a 15 ml Amicon© Ultra centrifugal filter unit (50 kDa MWCO, Merck Life Science) to about 130 mg ml^−1^ from an initial concentration of 2.3 mg ml^−1^. Protein concentration was estimated by UV absorption at 280 nm using the theoretical extinction coefficient calculated by ExPASy (Gasteiger et al., 2003).

### 4.2 Crystallization, data collection, and processing

As BpaC^741–1054^ is extremely soluble, initial crystallization screens for BpaC^741–1054^ were set up using about 130 mg ml^−1^ of protein using the sitting‐drop vapor‐diffusion method with a drop volume of 200 nl, the protein‐to‐reservoir ratio of 1:1 and a reservoir volume of 25 μl at 20 °C. Crystals grew readily in several conditions after 1 day in the JCSG screens I‐IV (NeXtal Biotechnologies, USA). We harvested crystals from 0.1 M HEPES pH 6.5, 0.8 M (NH_4_)_2_SO_4_. Here, 400 nl of cryo protectant buffer (0.15 M HEPES pH 6.5, 1.2 M (NH_4_)_2_SO_4_, 35% Glycerol) was added directly to the drop and incubated for 1 min before transferring to a liquid nitrogen container for data collection.

A total of 3600 images were collected by oscillation method with a range of 0.1 ° per image on a Dectris Eiger2 XE 16 M detector using single‐wavelength synchrotron radiation on beamline I04 at Diamond Light Source (Didcot, UK). Image processing was performed using XDS (Kabsch, 2010). Only images in the range of 800 to 2300 were used for data processing due to large changes in the *c* axis during the first 800 images, and the final dataset had a completeness of 99.5% to a resolution of 1.4 Å (Table 1).

### 4.3 Structure solution and refinement

Data reduction was performed with AIMLESS (Evans, 2011; Evans & Murshudov, 2013) and the model for molecular replacement (MR) was selected by using the sequence of BpaC^741–1054^ in the Advanced Search of the Protein Data Bank (PDB; Burley et al., 2019) and using the top hit (3S6L) for molecular replacement. The model was prepared using CLUSTALW (Larkin et al., 2007) to obtain a sequence alignment and CHAINSAW (Stein, 2008) to retain the conserved residues and truncate non‐conserved residues to alanine. The output model was used in a molecular replacement search using PHASER (McCoy et al., 2007) and then passed on to Buccaneer (Cowtan, 2006, 2012) for automated model building. This initial model was improved with several rounds of REFMAC5 (Murshudov et al., 2011) and manual model building in Coot (Vers. 0.8.9.3; Emsley et al., 2010), and the quality of refinement was checked against the MolProbity online server (Williams et al., 2018). Final refinement steps (Table 1) were done in PHENIX (Vers. 1.18‐3845‐000; Adams et al., 2011), and the final R‐factors were (R_work_/R_free_) 18.39%/21.71%.

### 4.4 Structure analysis and creation of a full C‐terminal head domain model

The structure was analyzed and images were created using PyMOL (Vers. 2.4.1; Schrodinger, 2017) and Inkscape (Version 1.0.1, https://inkscape.org). A full C‐terminal head domain, BpaC^431–1054^, was created by aligning layers in the solved structures in PyMOL and stacking them on top of each other. The term “layer” refers to a single 14‐long residue repeat within the C‐terminal head domain of BpaC with the conserved glycine at position 8 of the 14 residues that are present in all LPBRs. This was possible because the sequences between layers in the remaining C‐terminal head domain are identical to the first two layers of BpaC^741–1054^. This process is repeated until T431 and then merged in Coot. Varying side chains are replaced using the actual sequence and the side‐chain geometry information is taken from the corresponding layers in the actual structure which had the same residue at that position in the repeat. Size estimation of homology models and the extended head domain model (T731‐Q1054) was performed in PyMOL. APBS Electrostatics plugin in PyMOL was used for electrostatic surface visualization (https://pymolwiki.org/index.php/APBS). The angle between sheets was calculated using the psico plugin (https://pymolwiki.org/index.php/Psico) and angle_between_domains, using each layer as a single domain.

### 4.5 Sequence analysis and comparison with other trimeric autotransporters

The sequence of BpaC (https://www.uniprot.org/uniprot/A0A0H3HIJ5) was annotated using a combination of results from PSIPRED (Buchan & Jones, 2019), daTAA (Szczesny & Lupas, 2008), DeepCoil (Ludwiczak et al., 2019), Clustal Omega (Madeira et al., 2019), and well‐defined TAA structure motifs (Bassler et al., 2015; Kiessling et al., 2020). Alignment of LPBR head repeats (G742‐S1021) was performed by designation of the start and end point of each individual repeat, allowing comparison of residues within the repeats and the creation of a frequency plot using the WebLogo server (Crooks et al., 2004). Similar TAA structures were identified using a shortened structure model (S741‐A782, three repeats) as input for the Dali server (Holm & Laakso, 2016). Top hits that belonged to the TAA protein class were assessed and structurally compared using PyMOL. Frequency plots were created by following a similar pattern as for the BpaC C‐terminal head domain logo motif: First, the start and the end of the domain were identified in PyMOL; second, the glycine at position 8 of the usually 14‐residue long repeats was aligned by calculating the sequence length between glycines and identifying the correctly spaced ones (usually 14 residues), and breaking the sequence between residue 14 and 1 of the next layer. Alignments were cross‐checked with the actual structure. In the special case of the UspA1 LPBR (3PR7; Agnew et al., 2011) most layers consist of a 15‐residue repeat with an additional conserved glycine at position 9, which led to a different logo than for the other LPBRs. Residues in loops outside the 14‐residue core motif were not included in the frequency plots for the remainder of the LPBRs. These are still included in later calculations of solvent‐accessible residues and in the electrostatic surface presentation.

### 4.6 Homology analysis

We identified potential BpaC homologs using BpaC^741–1054^ in a PSI‐BLAST search against the NCBI database (Altschul et al., 1997) and a pBLAST (Altschul et al., 1990) against the *Burkholderia* genome database (Winsor et al., 2008). Prior to alignment, we removed all hits that did not provide at least 50% coverage of the query, were partial sequences, or were “obsolete” entries. The remaining sequences were aligned initially using MAFFT (Madeira et al., 2019) followed by manual curation in Jalview (Waterhouse et al., 2009) of the TAA head domain repeats. We deemed sequences lacking 14‐residue periodicity, G@8, and an additional fully conserved G@1 not BpaC‐like and discarded them from the analysis. The final sequences in alignment were used to construct a BpaC phylogenetic tree in MEGA X (Kumar et al., 2018). Default parameters for maximum‐likelihood methods in MEGA X were applied (Kumar et al., 2018) and the result was viewed using the interactive tree of life (iTOL) (Letunic & Bork, 2019).

### 4.7 Phylogenetic analysis and identification of pathogenicity islands

Sequences for BoaA, AtaA, EibD, UspA1, YadA, and “Adhesin YadA‐like (BoaC),” a presumed BpaC homolog from *B. oklahomensis*, were trimmed and manually aligned to BpaC^741–1054^. Alignments were generated in Jalview (Waterhouse et al., 2009) by designating G434 of BpaC as the start point for periodic repeats, as it contains a completely conserved Glycine at position 8. All other sequences were aligned to this one and gaps were introduced for repeats that deviated from the 14‐residue motif. Genomic islands for *bpaC* and *boaC* were predicted using IslandViewer4 (Bertelli et al., 2017). Functional annotation was acquired through InterPro (Blum et al., 2021). For the remaining TAA genes, adjacent genes were compared using a BLAST search against the KEGG database (Kanehisa & Goto, 2000).

### 4.8 Molecular dynamic simulations of solvent hydration maps

Atomistic MD simulations were performed using the crystal structure of BpaC^741–1054^ as input. All atomistic MD simulations used the AmberTools21 and Amber20 suite of programs (Case et al., 2021) with the FF14SB forcefield (Maier et al., 2015) used to describe the protein. The experimentally derived structures were protonated according to the Amber residue templates and then solvated with TIP3P water molecules in an octahedral box that extended 12 Å from the protein. Potassium ions were added to neutralize the system, then potassium chloride was added to a final concentration of 150 mM. After an initial energy minimization the system was heated to 300 K as positional restraints were decreased from 100 to 0 kcal mol^−1^ Å^−2^. Two unrestrained MD simulations were each performed for 1 μs starting from different arbitrary initial velocity distributions. The MD simulations used the pmemd.cuda module from Amber20 and were run on V100 GPUs. Hydration density maps were calculated using the ccptraj module within Amber20 using a grid spacing of 1 Å and a grid box size of 200 Å^3^ and saved relative to a time‐averaged pdb file of the protein calculated over the trajectory. The hydration densities were normalized relative to bulk water (1 g cm^−3^) and saved as XPLOR files for visualization in PyMOL. We used a density cutoff of seven times that of bulk water as it provides a good tradeoff between signal and background noise.

## AUTHOR CONTRIBUTIONS

Andreas R. Kiessling designed and carried out all wet‐lab experiments, structure building/analysis, and gene cluster analysis. Kathleen M. Weimer performed homology and phylogenetic analysis. Sarah A. Harris and Geoffrey Wells performed molecular dynamic simulations. Andreas R. Kiessling, Adrian Goldman, Sarah A. Harris, Kathleen M. Weimer and Geoffrey Wells wrote the manuscript. Adrian Goldman provided scientific input. All authors read and approved the manuscript.

## CONFLICT OF INTEREST

The authors declare that they have no conflicts of interest.

## ETHICAL STATEMENT

All GMO work was done in BSL 1 bacteria, according to best practice at the University of Leeds, and the University and laboratory have all appropriate licenses for this kind of work.

## Supporting information


**Appendix S1** Supplementary Information

## Data Availability

Model coordinates and associated structure factors of BpaC^741–1054^ were deposited at the Protein Data Bank in Europe (PDBe); accession code 7O23.
