# Supplementary material for: The C‐terminal head domain of Burkholderia pseudomallei BpaC has a striking hydrophilic core with an extensive solvent network
Source: Mol Microbiol. 2022 Jul 1;118(1-2):77–91. doi: 10.1111/mmi.14953 (PMC9543794; doi:10.1111/mmi.14953)
Supplement: Supplementary file 1 — Appendix S1 Supplementary Information [file MMI-118-77-s001.pdf]

Supplementary Information

Supplementary Materials and Methods

Structure prediction of LPBRs with unknown model using Robetta

The N-terminal head domain of AtaA (UniProt K7ZP88, residues 110-265) and BoaA (UniProt Q3JFX2, residues 180-437), for which there is no available structure were submitted to the Robetta server for structure prediction [1]. Residues with more than 2 Å local error estimate were omitted from the final model. Monomers were superimposed in PyMOL [2] onto the BpaC model with an equal number of LPBR layers and merged, thereby creating a trimeric model. Electrostatic surface visualisation was performed using the APBS plugin in PyMOL [2].

Table S1 Structural parameters of left-handed parallel β rolls - Angles and rises per repeat were calculated by superimposing 14-residue repeats centred on the completely-conserved G@8, and the PyMol plugin psico and "angle_between _domains" for calculation. The angles for UspA1 deviate significantly from the canonical values, but the structure as analysed by MolProbity is significantly poorer than the rest.

	BpaC					BoaA		AtaA		EibD		UspA1		YadA	
	Layer#	angle (°)	rise (Å)		Layer#	angle (°)	rise (Å)	angle (°)	rise (Å)	angle (°)	rise (Å)	angle (°)	rise (Å)	angle (°)	rise (Å)
	23	6.21	4.73	GSN	1										
	24	6.97	4.72	GDN	2	6.86	4.66	2.46	4.93	7.09	4.46	2.16	4.78	5.93	5.23
	25	6.34	4.87	GEN	3	6.71	4.53	4.24	4.84	4.58	5.03	2.47	4.82	5.87	4.68
	26	6.14	4.69	GSN	4	6.13	5.01	4.27	4.95	8.07	5.33	1.62	4.76	5.69	4.42
	27	6.01	4.73	GDN	5	5.52	4.69	3.03	4.72	4.43	5.06	1.56	4.95	6.62	4.96
	28	6.01	4.73	GEN	6	4.56	4.68	2.56	4.75	4.33	4.9	1.69	4.75	5.16	5.06
	29	5.70	4.88	GSN	7	3.92	5.27	3.23	5.05	2.31	5.04	2.6	4.89	4.89	5.39
	30	5.56	4.62	GDN	8			6.79	5.72			2.6	4.85	4.87	5.36
	31	5.36	4.83	GEN								2.73	5.08		
	32	6.15	4.89	GSN								2.18	4.59		
	33	6.03	4.93									6.46	4.73		
	34	4.98	4.73									5.61	5.24		
	35	5.20	4.89									7.95	5.93		
	36	6.56	4.48	GEN								4.18	4.41		
	37	5.26	4.86												
	38	5.28	4.98												
	39	4.89	4.91												
	40	5.82	4.65												
	41	4.48	4.93												
	42	2.45	4.81												
Mean		5.57	4.79			5.62	4.81	3.80	4.99	5.14	4.97	3.37	4.91	5.58	5.01
SD		0.93	0.12			1.08	0.25	1.39	0.31	1.91	0.26	1.97	0.36	0.59	0.33
Total		103.8	89.5			32.8	28.6	21.9	34.6	30	28.9	150	62	37.2	34.1

Table S2 Primers used to assemble the BpaC C-terminal Head domain for expression in *Escherichia coli.* Underlined sequences represent homologous parts required for three-fragment Gibson assembly with NEBuilder® HiFi DNA Assembly (New England Biolabs).

**Primer name**	**Sequence**
**bpaCCHead -r**	<u>TTATCTTCGATCTGTTTCAT</u>CTGGCCGACGTTGACGGCG
**bpaCCHead -f**	<u>TAAGAAGGAGATATACCATG</u>AGTGGCTCCAACTCAACCGCG
**pET28a -r**	<u>CATGGTATATCTCCTTCTTA</u>AAGTTAAAC
**pET28a -f**	<u>AAACACCACCACCAC</u>CACCAC
**RearGCN4 -r**	<u>GTGGTGGTGGTGTTT</u>AATCAGTTTTTAATACGCGCAATTTC
**RearGCN4 -f**	<u>ATGAAACAGATCGAAGATAA</u>AATTGAAGAAATCCTG

Supplementary Figures

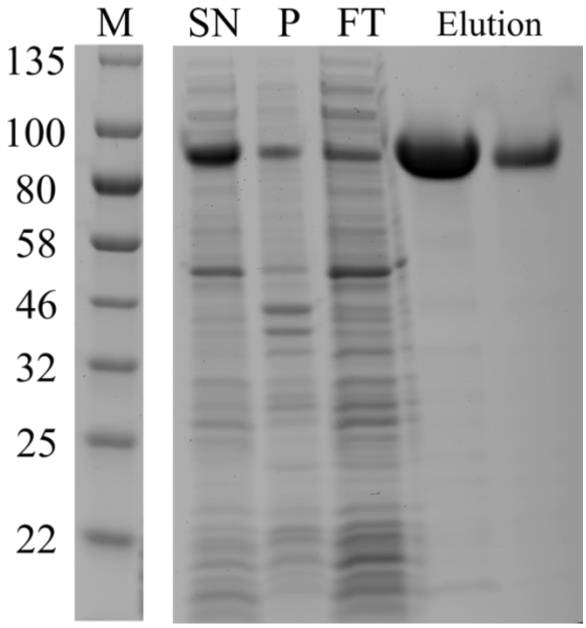

Figure S1 Coomassie stained SDS-PAGE of purified BpaC$^{741\text{-}1054}$. Samples from different stages of the IMAC purification process of BpaC$^{741\text{-}1054}$ are shown alongside a molecular mass marker (M). Supernatant (SN) and pellet (P) from the lysis step and flowthrough (FT) from IMAC have been loaded next to the IMAC elution (Elution). Expected monomer size is about 32 kDa with a clear trimer band for BpaC$^{741\text{-}1054}$ in the elution lanes.

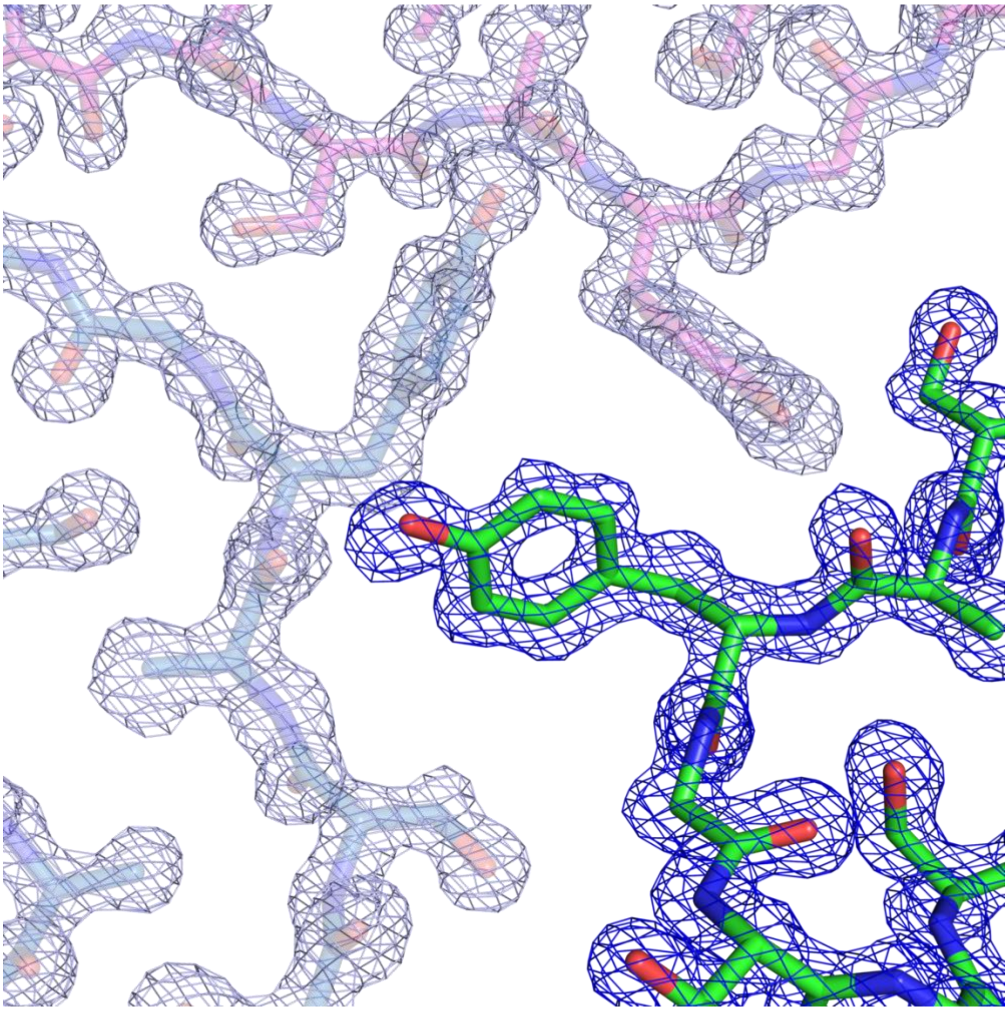

Figure S2 BpaC LPBR layer with electron density map. Selected layer of BpaC$^{741\text{-}1054}$ (G980-T993) with the central Y@7 shown in stick representation. Monomer "A" highlighted in green and the remaining monomers indicated in transparent view. Electron density map ($2F_o$-F_c) is displayed as blue mesh with a contour level of 1.5 σ.

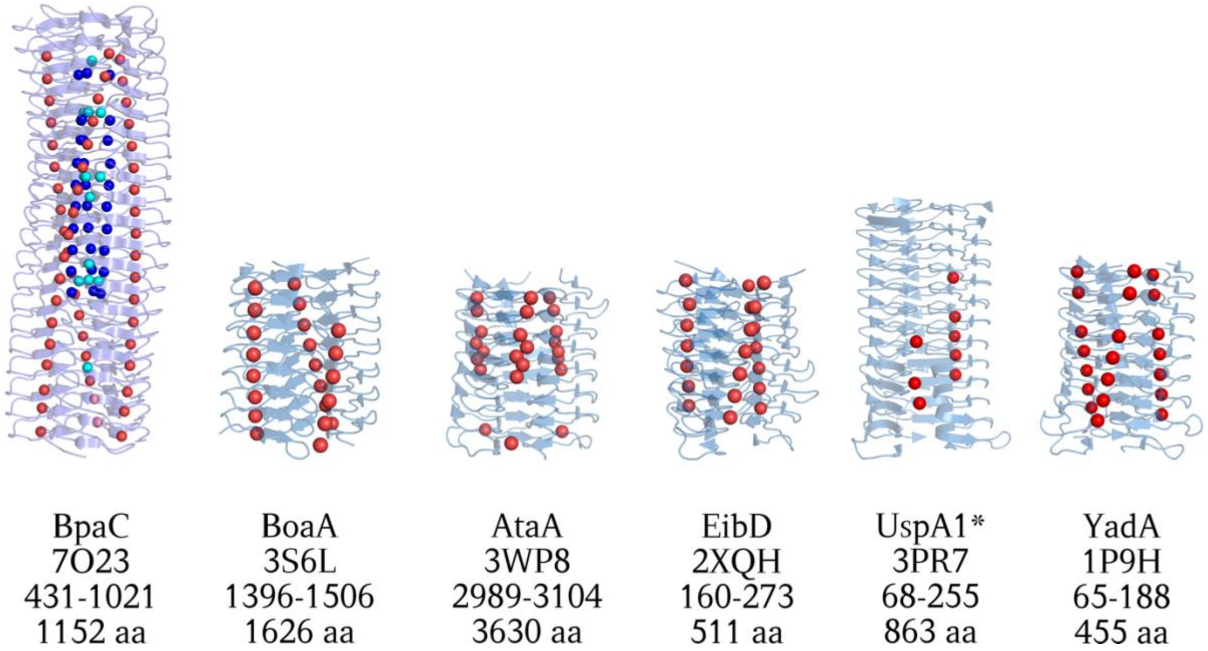

Figure S3 Comparison of conserved outer solvent channel in LPBRs. The highly conserved outer solvent channel (red spheres) is visible in all LPBR structures and in every layer. Exceptions to this are almost certainly due to resolution or model building mistakes. Especially in the electron density difference map (F_o-F_c) of UspA1 (3PR7), there is clear positive density for multiple solvent molecules along the outer solvent channel line that have not been built in the final model (not shown). Additional solvent molecules for BpaC$^{741\text{-}1042}$ are highlighted as inner solvent channel (blue spheres) and central solvent molecules (cyan spheres). Protein name, PDB ID, displayed residue range, and full protein length are shown.

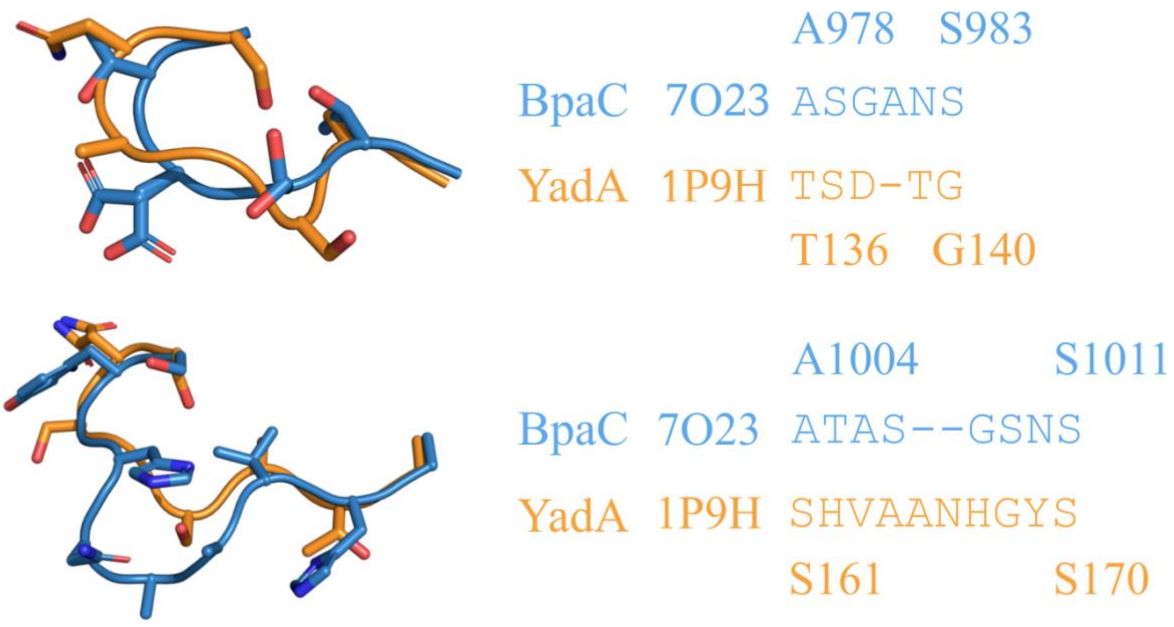

Figure S4 Comparison of loops of BpaC$^{741-1054}$ and YadA head to demonstrate effect of loops. The most common repeat length of 14 can deviate by having a longer or shorter loop between layers. This does not influence the Cα position of core residues of the layer that before or after the unusual loop. A comparison of normal loop length and a shorter loop (top) and longer loop (bottom) is shown. Chain trace of the loops of selected layers of BpaC$^{741-1054}$ (blue) with their corresponding loops of unusual lengths in YadA with PDB code and residue positions (orange).

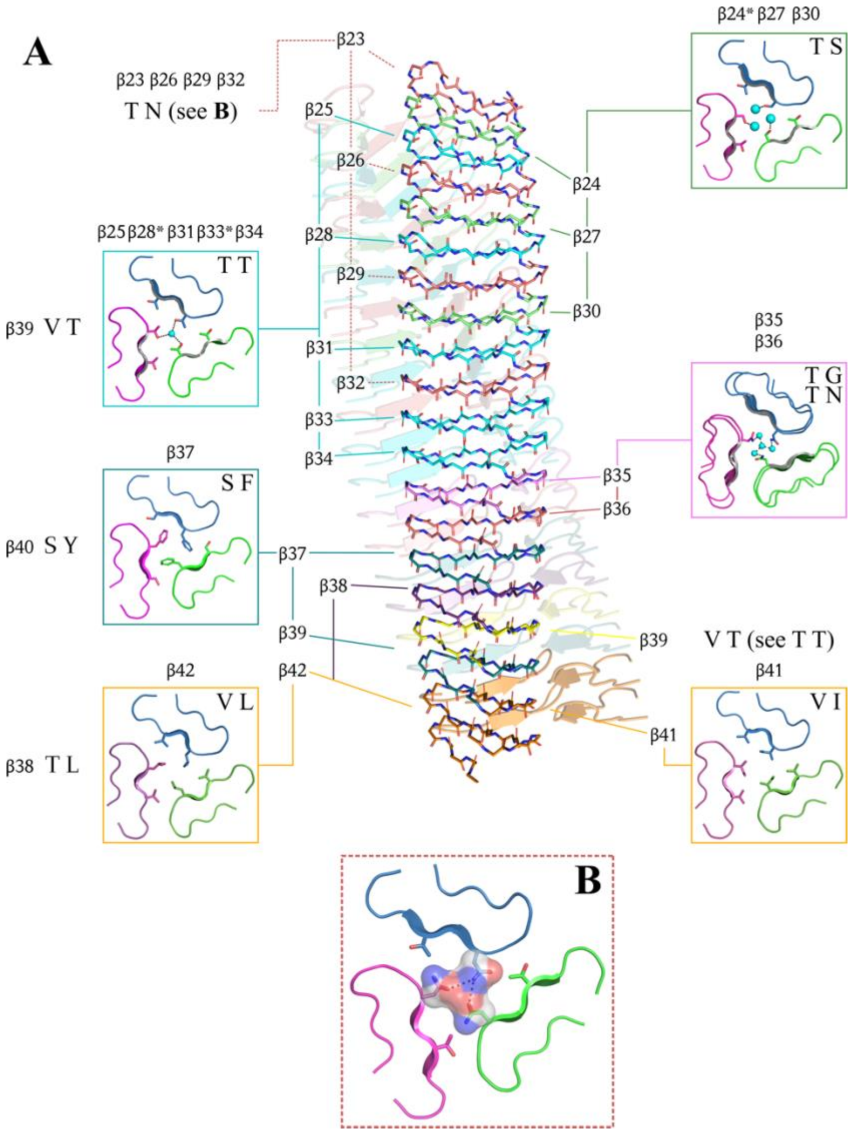

Figure S5 Overview of all LPBR core motifs in BpaC$^{741\text{-}1042}$. (**A**) BpaC shown as sticks and coloured in groups of similar motifs to better show the distribution between repeats. Individual layers are grouped by similar residues at position 7/14 with their respective layer number given next to the model of the whole head domain and the individual motifs. Sidechains for position 5+7/14 are shown along with central solvent molecules (cyan spheres) and H-bonds. Letters: residues at position 5 and 7 for each layer. Motifs with missing central solvent molecules (S/T@7) are labelled with an asterisk (*). (**B**) Zoomed-in view of one of the most frequent LPBR core motifs: T@5 and N@7 shown with atom volume and electrostatic charge for N@7. We propose that, to stabilise the core, one asparagine sidechain is flipped as shown, but this is not stable in refinement as it requires multiple coordinated occupancies of 1/3.

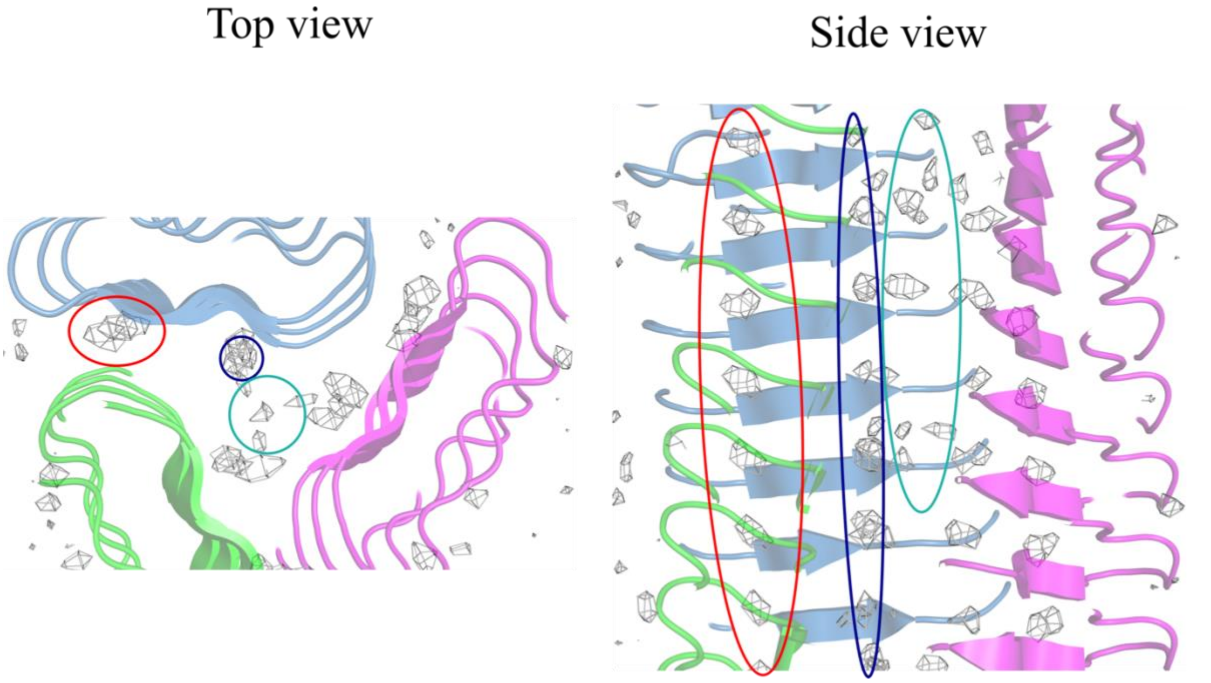

Figure S6 Overlay of hydration density maps and crystal structure of BpaC$^{741-1054}$. Averaged hydration density map cutoff was normalised and set at 7x relative to bulk water. XPLOR files were imported into PyMOL and overlayed onto the PDB file that was generated as averaged output from the two MD simulation repeats. Only a few layers of the total structure are shown here to better visualise the individual hydration density peaks. Density peaks that correlate with outstanding solvent molecules previously identified in the crystal structure have been highlighted as circles: outer solvent channel (red), inner solvent channel (blue), central solvent molecules (cyan).

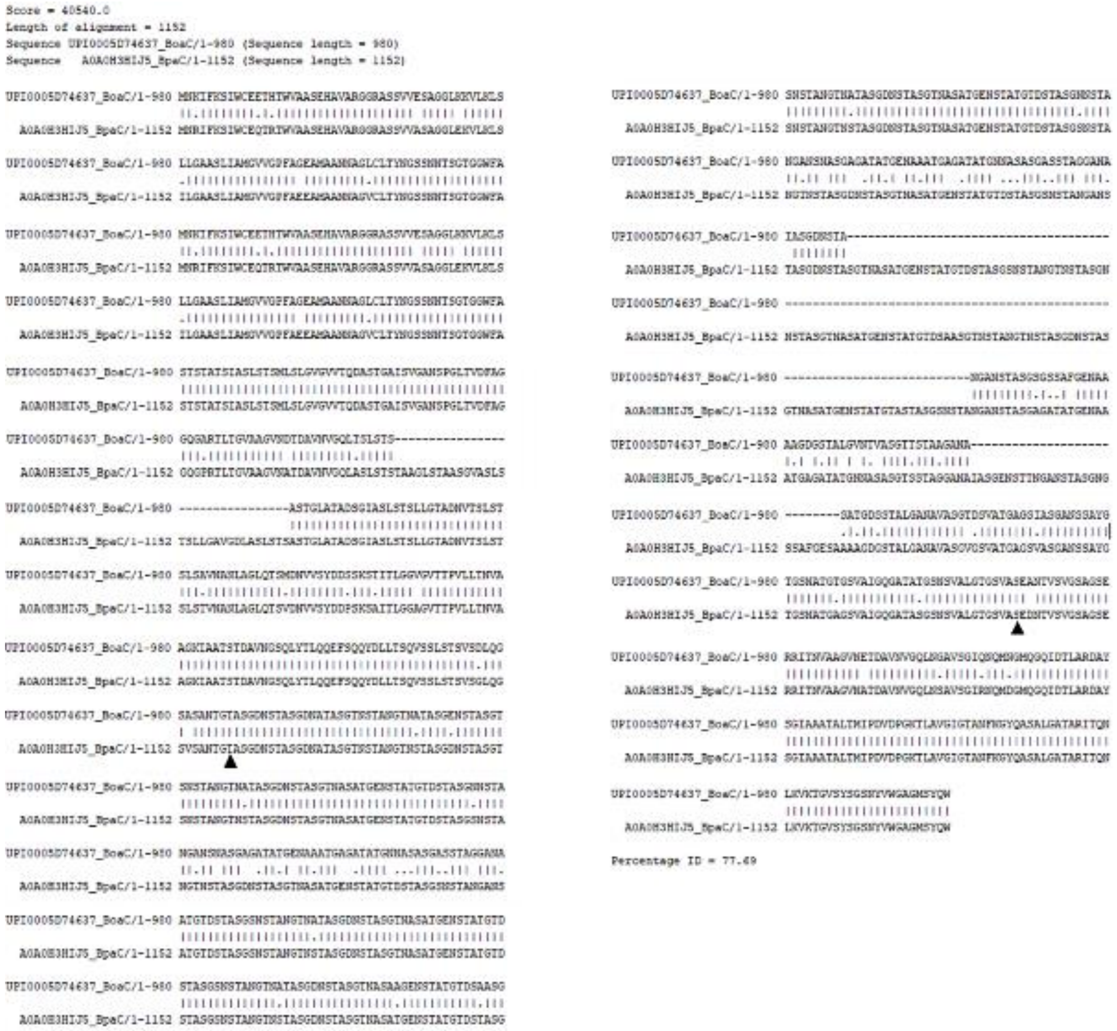

Figure S7 Pairwise alignment of full length BpaC with the homologue BoaC. Black arrows indicate the start and end of the C-terminal BpaC head sequence (431-1021).

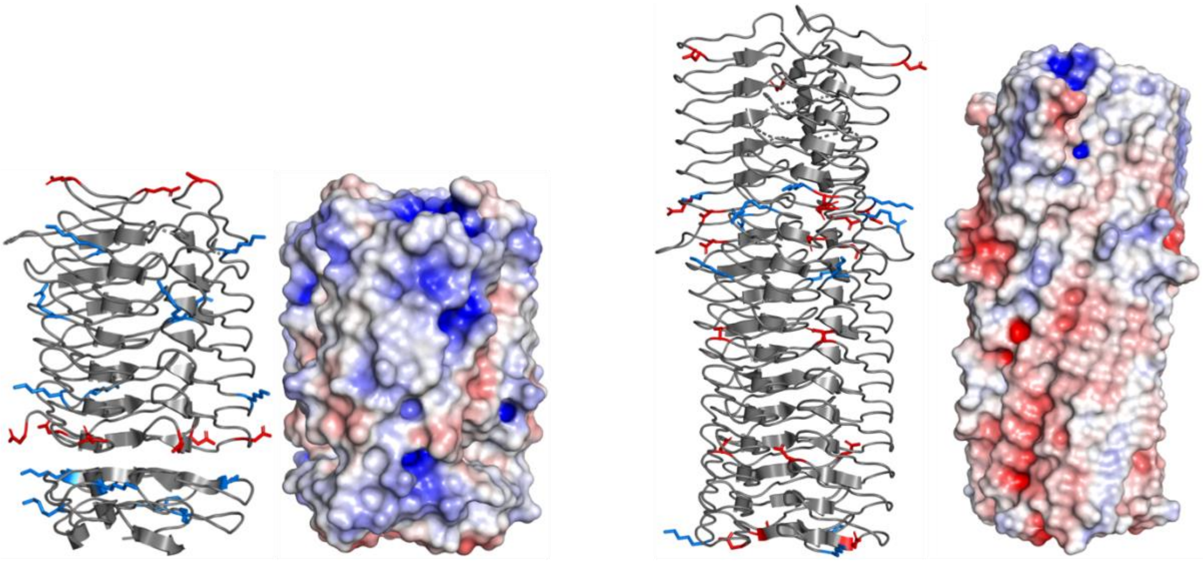

AtaA
K7ZP88
110-265
3630 aa
Δ120-124
Δ219-234

BoaA
Q3JFX2
180-437
1626 aa
Δ224-230
Δ244-248

Figure S8 Surface charge representation of Robetta [1] predicted models of AtaA and BoaA. Residues carrying charges at pH 7 are highlighted in colour and electrostatic surface charge is shown created by the APBS plugin in PyMOL [2]. Name, UniProt ID, residue range used as input for Robetta, total protein length, and deleted segments due to local error threshold is shown.

Supplementary References

[1] M. Baek *et al.*, "Accurate prediction of protein structures and interactions using a three-track neural network," *Science*, vol. 373, no. 6557, pp. 871-876, Aug 20 2021, doi: 10.1126/science.abj8754.

[2] L. Schrodinger, "The PyMOL Molecular Graphics System, Version 2.0," ed, 2017.
